# FEASIBILITY AND PRELIMINARY EFFICACY OF THE KINDER INTERVENTION TO PROMOTE HEALTHY CARE RELATIONSHIPS

**DOI:** 10.1093/geroni/igae098.0933

**Published:** 2024-12-31

**Authors:** Kylie Meyer, Jeanine Yonashiro-Cho, Sohee Kim, Donna Benton

**Affiliations:** Case Western Reserve University, Cleveland, Ohio, United States; University of Southern California Keck School of Medicine, Los Angeles, California, United States; Case Western Reserve University, Cleveland, Ohio, United States; University of Southern California, Los Angeles, California, United States

## Abstract

Knowledge and Interpersonal Skills to Develop Enhanced Relationships (KINDER) is a psychoeducational intervention designed to prevent emotional-type elder mistreatment by family caregivers to persons living with dementia. Informed by evidence-based approaches in intimate partner violence, caregivers in this virtual 8-week course develop disease knowledge, communication strategies, and coping skills to manage sources of relationship strain. Components include facilitated group Zoom discussion sessions and asynchronous weekly readings and practice exercises, including story-based videos informed by caregiver focus groups. In this pre- and post-test study, we examine KINDER’s feasibility and preliminary efficacy. A total of N=45 caregivers enrolled from March 2023 to January 2024. Among participants, n=39 have already completed all data collection activities, including n=4 who were lost to follow-up and n=3 who withdrew (82% retention). The primary reason for withdrawal was believing the program was not relevant to their care situation. On average, study participants attended 2.4 of 3 Zoom discussion sessions (SD=0.7). Seventy-eight percent (78.1%) of participants indicated they completed asynchronous activities “all” (40.6%) or “most” (37.5%) of the time. Findings from paired t-test analyses of outcomes indicate a reduction in potentially harmful care behaviors (e.g., threatened to send family member to a nursing home; p=0.025). Consistent with the goal of the intervention, caregivers also reported improved relationship quality with the care recipient (p<0.001). All program completers indicated they were “very satisfied” (77.4%) or “satisfied” (22.6%) with KINDER. Findings from this pilot study demonstrate KINDER’s feasibility. Future research will rigorously test intervention efficacy and examine likely mechanisms of change.

